# Pulmonary interstitial emphysema presenting in a woman on the intensive care unit: case report and review of literature

**DOI:** 10.1186/1752-1947-5-236

**Published:** 2011-06-25

**Authors:** Peter B Sherren, Tomas Jovaisa

**Affiliations:** 1 Department of Anesthesia and Intensive Care, Queen's Hospital, Rom Valley Way, Romford, Essex, RM7 0AG, UK

## Abstract

**Introduction:**

Pulmonary interstitial emphysema is a life-threatening form of ventilator-induced lung injury. We present one of the few reported adult cases of pulmonary interstitial emphysema in a woman with respiratory failure admitted to our intensive care unit.

**Case presentation:**

An 87-year-old Caucasian woman with a diagnosis of community-acquired pneumonia was admitted to our intensive care unit requiring invasive ventilation. The combination of a poor oxygenation index and bilateral alveolar/interstitial infiltrates on a chest radiograph fulfilled the criteria for adult respiratory distress syndrome; the cause was thought to be a combination of the direct pneumonic pulmonary injury and extrapulmonary severe sepsis. By day seven, the fraction of inspired oxygen, peak airway and positive end expiratory pressures weaned sufficiently to allow an uncomplicated percutaneous tracheostomy. On day 10, problems with ventilation necessitated recruitment maneuvers with a Mapleson C circuit, after which dramatic surgical emphysema was noted. An upper airway bronchoscopy showed no obvious tracheal wall injury, and computed tomography of her chest showed extensive surgical emphysema, perivascular emphysema and peribronchial emphysema, which were consistent with a diagnosis of pulmonary interstitial emphysema. Over the following days, despite protective ventilatory strategies and intercostal tube thoracostomy, lung compliance along with oxygenation deteriorated and our patient died on day 14.

**Conclusion:**

The development of pulmonary interstitial emphysema is a rare but real risk when caring for patients with worsening lung compliance on the intensive care unit. Improved awareness of the condition, early protective ventilation strategies and timely treatment of any of the lethal complications will hopefully result in improved survival from the condition in adults.

## Introduction

Pulmonary interstitial emphysema (PIE) is a barotrauma-related life-threatening condition, not uncommon to the neonatologist caring for pre-term babies. For the intensivist, despite being confronted by significant compliance issues resulting from the fibroproliferative phase of adult respiratory distress syndrome (ARDS) on a daily basis, PIE in the critically ill adult is an extremely rare occurrence. The infrequency of PIE on the intensive care unit means that recognition of the condition, knowledge of its clinical sequelae, and its management among physicians is poor. We present the case of a woman admitted to our unit, who developed PIE, and died. We believe this to be one of the only few reported adult cases of barotrauma-related PIE.

## Case presentation

**Figure 1:**
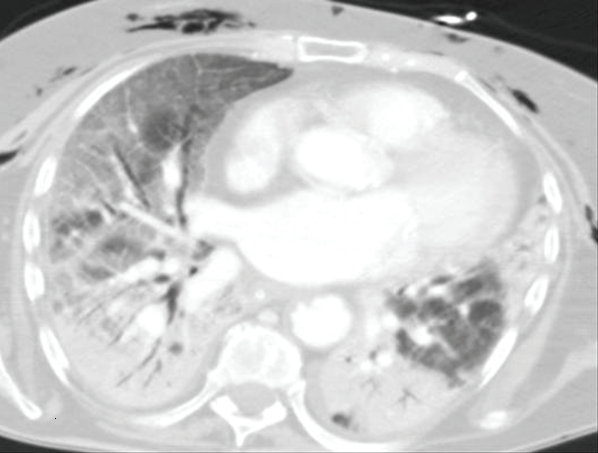
**Axial CT chest scan**.

An 87-year-old Caucasian British woman presented to our emergency department with a three-day history of shortness of breath, pyrexia and non-productive cough. Her only significant past medical history was well controlled hypertension. She was independent in her daily activities, did not smoke cigarettes and reported a good cardiorespiratory reserve prior to the onset of symptoms. The diagnosis of community-acquired multilobar pneumonia was made with a CURB-65 score of three. She was admitted to the high dependency unit with type 1 respiratory failure and a high alveolar-arterial oxygen gradient. She received intravenous antibiotics (piperacillin/tazobactam and erythromycin) and non-invasive high-flow continuous positive airway pressure (CPAP). By the fourth day, our patient had deteriorated, with a chest radiograph showing bilateral alveolar and interstitial infiltrates and a ratio of partial pressure of arterial oxygen to the fraction of inspired oxygen (PaO_2_/FiO_2_) of < 26.6 kPa, which required invasive ventilation. The criteria for ARDS were met and the cause was thought to be a combination of the direct pneumonic pulmonary injury and extrapulmonary severe sepsis. With protective lung ventilation, low dose methylprednisolone, antibiotic therapy and a negative fluid balance, gradual improvement was made; over the following five days respiratory parameters weaned sufficiently to allow an uncomplicated percutaneous tracheostomy to be performed. On day 10, a period of desaturation required recruitment maneuvers with a Mapleson C circuit that resulted in notable surgical emphysema. The cause was thought to be a tracheal injury sustained at the time of tracheostomy insertion. An adjustable flange tube was positioned under bronchscopic guidance just proximal to the carina in an attempt to limit any further tracking of air through the potential tracheal defect. Despite these measures, a high alveolar-arterial oxygen gradient and peak airway pressures persisted. A chest radiograph showed more homogenous central pulmonary alveolar shadowing. An upper airway bronchoscopy showed no obvious tracheal wall injury and computed tomography (CT) of her chest showed extensive surgical emphysema and a small anterior left sided pneumothorax. On further review of the CT scan, it was felt that the perivascular and peribronchial emphysema was consistent with a diagnosis of pulmonary interstitial emphysema (Figure 1). Over the following days, despite protective ventilatory strategies and intercostal tube thoracostomy, lung compliance along with oxygenation deteriorated. By day 13, the deteriorating respiratory parameters along with inotropic requirements resulted in a decision to limit therapy and the patient died on day 14.

## Discussion

In a large retrospective study, Greenough *et al*. found the incidence of PIE in pre-term infants requiring ventilation for respiratory distress syndrome to be around 19.5%, with a mortality rate of 24% [1]. These figures are known to be falling with the growing use of new ventilation strategies and availability of high frequency oscillatory ventilation. PIE almost exclusively occurs as a result of intermittent positive pressure ventilation (IPPV) with peak airway pressures exceeding 30 cm H_2_O [2]. When high airway pressures and dramatic shearing forces are applied to a non-compliant lung unit, the result is alveolar duct rupture, usually at the terminal bronchiole/alveolar junction [3]. This allows air to escape into the connective tissue of the peribronchovascular sheaths, interlobular septa and visceral pleura, occasionally migrating into the lymphatic and venous circulation [3]. The emphysema can be localized, unilateral or, as in our case, bilateral and diffuse. Pre-term babies are particularly prone to PIE, because of the high shearing forces and airway pressures required to re-recruit lung units with collapsing pressure exceeding their functional residual capacity, secondary to reduced surfactant levels [3]. Other postulated risks for PIE include increased amount of pulmonary connective tissue or a sudden reduction in extravascular lung water, which may offer a degree of protection against tracking interstitial emphysema. Poor lung compliance was pivotal in our case, but a reduction in extravascular lung water also perhaps had a role to play in the development of PIE.

Interstitial emphysema has a number of potentially detrimental sequelae [1,4]. These include: compression atelectasis of adjacent healthy lung and resulting intrapulmonary shunt which is worsened by recruitment maneuvers; compression of surrounding pulmonary vasculature; and decompression of interstitial blebs into surrounding spaces, potentially resulting in pneumomediastinum, pneumothorax, pneumopericardium, pneumoperitoneum and surgical emphysema.

Although all the above can be very difficult to manage in a critically ill patient, the addition of a pneumothorax to PIE alone doubles the mortality [1].

PIE is generally felt to be a radiographic and pathologic diagnosis, although the presence of recurrent pneumothoraces and large persistent alveolar-arterial gradient in the presence of high peak airway pressures should at least arouse a suspicion of its presence.

Chest radiograph findings are often very subtle, and identification, given the frequent presence of overlying dense alveolar shadowing as a result of the lung injury and exudative processes, make the diagnosis difficult. However, the following findings may sometimes be distinguishable [4-6]: parenchymal stippling; lucent mottling and streaking extending to the mediastinum; perivascular halos (from perivascular air collections); subpleural cysts; lucent bands; and parenchymal cysts or bullae.

CT is a more sensitive tool for delineating the pathology, and the classic findings of tracking perivascular and peribronchial emphysema [7] were both demonstrated in our case (Figure 1).

The chosen treatment will, to an extent, depend on the distribution of the disease along with the severity and complications. The mainstay of treatment is to achieve adequate oxygenation with lower mean and peak airway pressures, hence minimizing interstitial leak through the defects [8]. This technique of protective lung ventilation and permissive hypercapnia is a familiar one to intensivists trying to avoid the many ramifications of volutrauma and barotrauma. There are a number of other therapeutic options that may be considered. Lateral decubitus positioning with the affected lung in the dependent position can be tried as an early conservative approach, encouraging plugging of the dependent lung. This is only of benefit when the disease is localized. Selective main bronchial intubation and occlusion can be useful, although only for unilateral disease. High-frequency ventilation (high frequency jet ventilation or high frequency oscillatory ventilation) can also be effective. Finally, extracorporeal membrane oxygenation can be used [1-3,8].

Beyond these mainstays of treatment, there have been some case reports and series regarding the use of steroids and surgical resection for persistent localized disease, but such therapies have not established a good evidence base as yet.

Once the diagnosis of PIE is established, a high degree of vigilance must be maintained for any potentially serious complications, coupled with prompt and appropriate interventions.

## Conclusion

The meticulous employment of protective lung strategies throughout intensive care units has been a vital step towards avoiding volutrauma and/or and improving outcomes and survival in acute lung injury (ALI) and ARDS [9]. However, despite these measures, development of the fibroproliferative and fibrotic phases of ALI and ARDS are commonplace. The associated reduction in lung compliance produces a new range of challenges, which is distinct from the exudative and/or hypoxia phase faced in the early disease.

Recruitment maneuvers are commonly undertaken on the intensive care unit. They are vital for increasing the functional residual capacity above the closing capacity, and therefore providing improved lung compliance and oxygenation. When undertaking recruitment maneuvers and interpreting peak airway pressures, it is important to remember the differential lung time constants encountered throughout the diseased lungs. These variations result in an uneven distribution of pressure across the alveoli, and areas of lung with long time constants are at high risk of barotrauma. It is these areas that are at particular risk of developing PIE when exposed to the shearing forces experienced during IPPV.

The development of PIE is a rare but real risk when caring for patients with worsening lung compliance. Improved awareness of the condition, early protective ventilation strategies and timely treatment of any lethal complications will hopefully result in improved survival from the condition in adults.

## Consent

Written informed assent was obtained from the patient's next of kin for publication of this case report and accompanying images. A copy of the written consent is available for review by the Editor-in-Chief of this journal.

## Competing interests

The authors declare that they have no competing interests.

## Authors' contributions

TJ was involved in the care of the patient. PS and TJ analyzed the case history and CT scans. PS and TJ were involved in drafting the manuscript and both authors read and approved the final manuscript.
